# Crystal structure of the two-subunit tRNA m^1^A58 methyltransferase TRM6-TRM61 from *Saccharomyces cerevisiae*

**DOI:** 10.1038/srep32562

**Published:** 2016-09-01

**Authors:** Mingxing Wang, Yuwei Zhu, Chongyuan Wang, Xiaojiao Fan, Xuguang Jiang, Mohammad Ebrahimi, Zhi Qiao, Liwen Niu, Maikun Teng, Xu Li

**Affiliations:** 1 Hefei National Laboratory for Physical Sciences at Microscale, Innovation Center for Cell Signalling Network, School of Life Science, University of Science and Technology of China, Hefei, Anhui, 230026, People’s Republic of China; 2 Key Laboratory of Structural Biology, Hefei Science Center of CAS, Chinese Academy of Science, Hefei, Anhui, 230026, People’s Republic of China

## Abstract

The N^1^ methylation of adenine at position 58 (m^1^A58) of tRNA is an important post-transcriptional modification, which is vital for maintaining the stability of the initiator methionine tRNA_i_^Met^. In eukaryotes, this modification is performed by the TRM6-TRM61 holoenzyme. To understand the molecular mechanism that underlies the cooperation of TRM6 and TRM61 in the methyl transfer reaction, we determined the crystal structure of TRM6-TRM61 holoenzyme from *Saccharomyces cerevisiae* in the presence and absence of its methyl donor S-Adenosyl-L-methionine (SAM). In the structures, two TRM6-TRM61 heterodimers assemble as a heterotetramer. Both TRM6 and TRM61 subunits comprise an N-terminal β-barrel domain linked to a C-terminal Rossmann-fold domain. TRM61 functions as the catalytic subunit, containing a methyl donor (SAM) binding pocket. TRM6 diverges from TRM61, lacking the conserved motifs used for binding SAM. However, TRM6 cooperates with TRM61 forming an L-shaped tRNA binding regions. Collectively, our results provide a structural basis for better understanding the m^1^A58 modification of tRNA occurred in *Saccharomyces cerevisiae*.

Transfer RNA (tRNA), typically 76 to 90 nucleotides in length, is a necessary component of the protein translation machinery^1^,^2^. It serves as a carrier delivering amino acids to the ribosome for protein synthesis. An indispensable role of tRNAs is that they could specify which sequence from the genetic code corresponds to which amino acid^3^. In eukaryotes, tRNAs are transcribed by RNA polymerase III as pre-tRNAs. During tRNAs maturation, pre- tRNAs undergo a series of post-transcriptional modifications, including splicing, end- trimming, and base or ribose modifications. These modifications are helpful to stabilize the 3D structure of tRNAs, and are crucial to ensure the translation efficiency and fidelity^3^,^4^,^5^.

To date, 108 currently known chemical modifications of tRNA have been identified, Of these, the most common and prevalent tRNA modifications is methylation^6^. Adenine at position 58 (A58) located in the T-loop is one of the most conserved nucleosides in tRNA, and the occurrence of 1-methyladenosine (m^1^A) at position 58 has been reported from all three kingdoms^7^. This modification also plays an important biological role in both eukaryotes and prokaryotes. For example, tRNA m^1^A58 modification is required for *Thermus thermophilus* to survive at high temperatures^8^. In yeast, inactivation of gene coding for m^1^A58 tRNA methyltransferase causes the instability of initiator tRNA_i_^Met^, and tRNA_i_^Met^ lacking m^1^A58 modification is subsequently degraded by nuclear exosome^9^,^10^. Particularly important is that methylated adenine at position 58 in human tRNA_3_^Lys^ is closely associated with immunodeficiency virus type 1 (HIV-1). Studies have proved that m^1^A58 modification of tRNA_3_^Lys^ is required for both efficacy and fidelity of (+) strand DNA transfer during HIV replication^11^,^12^.

The RNA methyltransferases are responsible for this modification, which catalyze the transfer of methyl group from the methyl donor S-adenosyl-L-methionine (SAM) to various positions of the nucleotide bases, yielding a methylated RNA and S-adenosyl-homocysteine (SAH)^13^. All known RNA methyltransferases can be classified into four superfamilies, including Rossmann-fold, SPOUT, Radical-SAM and FAD/NAD(p)-dependent methyl- transferases^14^,^15^. The m^1^A58 tRNA methyltransferase is appropriate for the Rossmann-fold superfamily. Thus far, the crystal structures of four bacterial m^1^A58 tRNA methyltransferases have been reported, comprising MtTrmI (m^1^A58 tRNA methyltransferases from *Mycobacterium tuberculosis*)^16^,^17^, TtTrmI (m^1^A58 tRNA methyltransferases from *Thurmus thermophilus*)^18^, AaTrmI (m^1^A58 tRNA methyltransferases from *Aquifex aeolicus*), and PaTrmI (m^1^A58 tRNA methyltransferases from*Pyrococcus abyssi*)^19^,^20^. These studies indicate that the m^1^A58 tRNA methyltransferases from bacterial and archaeal consist of one subunit and function as homotetramers.

Distinctly different from bacterial and archaeal, the known eukaryotic m^1^A58 tRNA methyltransferases are composed of two subunits and supposed to function as heterotetramers. In yeast, the two subunits complex TRM6-TRM61 has been identified as the key factor of this methyl transfer reaction^21^,^22^. Study discloses that *S.cerevisiae* TRM6-TRM61 complex is vital for the m^1^A58 modification in the processing and accumulation of tRNA_i_^Met^, which is a key initiation step of translation^21^,^22^,^23^. Detailed sequence analysis further demonstrates that TRM61 possesses a typical Rossmann-fold, and shares obvious sequence similarity to the bacterial and archaeal m^1^A58 tRNA methyltransferase TrmI. In contrast, TRM6 homologs have been identified only in eukaryotes, and no evident sequence similarity with any proteins other than orthologs is found. This unique binary composition of eukaryotic m^1^A58 tRNA methyl- transferases hints a different mechanism for tRNA recognition and catalysis than its prokaryotic counterparts.

In this study, we report the crystal structures of *S.cerevisiae* TRM6-TRM61 holoenzyme and TRM6-TRM61 in complex with SAM. TRM6 and TRM61 adopt a similar fold, with a high structural similarity to the prokaryotic counterpart TrmI. Differently, the *S.cerevisiae* TRM6 and TRM61 evolve more structural elements to better suit the 3D conformation of target tRNA. Further, the structural comparison of the wild-type TRM6-TRM61 and TRM6-TRM61-SAM disclose the fine rearrangements of the active site upon SAM binding. When we prepared this manuscript, the structure of TRM6-TRM61-tRNA_3_^Lys^-SAM from *H. sapiens* was reported by Stroud’s group^24^. Here, our results provide a tRNA-unbound state TRM6-TRM61 holoenzyme, which assists in understanding the conformation change upon tRNA binding occurred in eukaryotes. Taken together, these two structures suggest a hand open-close strategy for eukaryotic tRNA m^1^A58 methyltransferases to recognize target tRNAs.

## Results and Discussion

### Overall structure of TRM6-TRM61 holoenzyme

**Figure 1:**
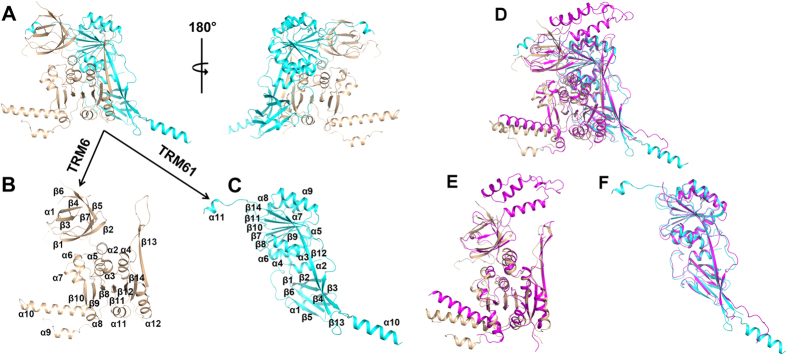
Overall structure of TRM6-TRM61 complex. (**A**) Two views of the complex structure of TRM6 with TRM61. TRM6 and TRM61 are colored in tint and cyan, respectively. (**B**) Cartoon show of the structure of TRM6. (**C**) Cartoon show of the structure of TRM61.α-helices and β-strands are labeled, respectively. (**D**) Superposition of *S. cerevisiae* TRM6-TRM61 with the *H. sapiens* homologue. *S. cerevisiae* TRM6 and TRM61 are colored the same as Fig. 1A. The *H. sapiens* TRM6-TRM61 complex is colored in magenta. (**E,F**) Structural superposition of *S. cerevisiae TRM6* (**E**) and TRM61 (**F**) with its *H. sapiens* counterparts. The TRM6 and TRM61 are colored the same as Fig. 1D.

To improve understanding the target tRNA recognition and catalytic mechanism of the two component m^1^A58 tRNA methyl- transferase in eukaryotes, we solved the structure of the TRM6-TRM61 holoenzyme from *S. cerevisiae* by the SAD method using the Se anomalous signal. The final model has been refined to 2.2 Å resolution and the details of the data collection and refinement statistics are summarized in Table 1. As shown in Fig. 1A, one TRM6-TRM61 heterodimer adopts a rectangular shape with two arms protruding from the core.

TRM61, the eukaryotic homologue of the bacterial and archaeal m^1^A58 tRNA methyl- transferase TrmI, consists of two distinct domains: a small N-terminal domain (residues 4–78) and a large C-terminal domain (residues 79–378). The small N-terminal domain consists of three hairpin β-motifs, which form a β-barrel (Fig. 1C). The large C-terminal domain adopts a typical Rossmann-fold, comprising a central seven-stranded β-sheet flanked by three α-helices on each side (Fig. 1C). The first five strands of the β-sheet are parallel, while the other two strands at the C-terminal end are antiparallel. Between α9 and β14, there is an arm protruding from the globular Rossmann-fold, including α10, antiparallel β12 and β13, and a highly flexible region spanning residues 286–331 (Fig. 1C). The N- and C-terminal domains are linked by α2 and stabilize each other via extensive hydrophobic interactions.

TRM6 contains 478 amino acid residues, comprising an N-terminal β-barrel domain (residues 1–196) and a C-terminal Rossmann fold domain (residues 197–451). Due to the flexibility, residues 61–78, residues 88–188, residues 322–325 and residues 453–478 are not observed. The N-terminal β-barrel domain consists of seven antiparallel β strands and a highly flexible region (~120 amino acid residues) that is not visible in the electron density. Similar to most Class I SAM-dependent methyltransferases, the C-terminal Rossmann-fold of TRM6 is also composed of a central seven-stranded β-sheet flanked by α-helices on both sides. With some changes, there is an arm insertion between β10 and β11, including α8, α9 and α10 (residues 302–360) (Fig. 1B). TRM6 presented in our structure displays an overall similar 3D structure with TRM61. A Dali research has further confirmed that TRM6 is structurally homologous to the TrmI family proteins, such as MtTrmI (PDB code 1I9G), TtTrmI (PDB code 2PWY), PaTrmI (PDB code 3MB5) and AaTrmI (PDB code 2YVL), with a Dali Z-score of 21.5, 20.5, 19.1 and 18.7, respectively.

When we launched rigorous trials aimed at crystallizing TRM6-TRM61 in complex with the target tRNA_i_^Met^, the complex structure TRM6-TRM61-tRNA_3_^Lys^-SAM from *H. sapiens* was resolved by Stroud and his colleague^24^. The yeast TRM6-TRM61 shares a sequence similarity with the human homologue (Figure S1–2). The TRM6 and TRM61 in *S. cerevisiae* possess 20.5% and 30.2% identity with that in *H. sapiens*, respectively. The overall structure of TRM6-TRM61 in *S. cerevisiae* adopts a similar fold as that in *H. sapiens* (Fig. 1D). The overall main-chain r.m.s.d between the *S. cerevisiae* and *H. sapiens* TRM6 is 2.07 Å for 156 comparable C_α_ atoms, and the overall main-chain r.m.s.d between the *S. cerevisiae* and *H. sapiens* TRM61 is 1.03 Å for 211 comparable C_α_ atoms (Fig. 1E,F).

### The heterodimer interface of TRM6-TRM61

**Figure 2:**
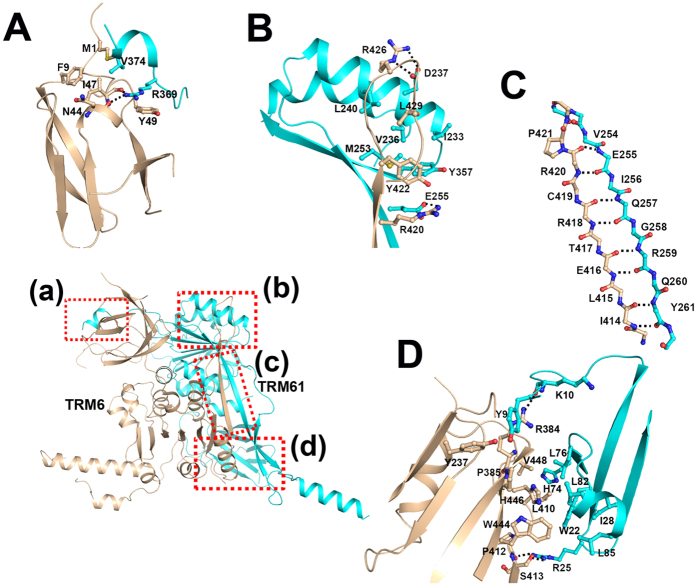
The heterodimer interface of TRM6-TRM61. The interface of TRM6 and TRM61heterodimer can be divided into four sites. (**A**) Details show of the interaction between the C-terminal helix α11 of TRM61 and the N-terminal β-barrel domain of TRM6. (**B**) Cartoon show of the interface between the “n” shape loop following strands β13 of TRM6 and the concave surface enclosed by strand β12 and α9 of TRM61. (**C**) Cartoon show of the network of hydrogen bonds in the β-sheet formed by the strand β12 of TRM6 and the strand β13 of TRM61. (**D**) Details show of the interaction between the N-terminal β-barrel domain of TRM61 and the Rossmann fold domain of TRM6. The residues involved in the interaction are labeled and shown as sticks, hydrogen bonds are indicated by dashed lines.

TRM6 and TRM61 form a compact complex via numerous hydrogen bonding and extensive hydrophobic interactions. The heterodimer interface of TRM6-TRM61 buries 3194 Å^2^ of TRM6 and 3167 Å^2^ of TRM61 solvent- accessible area, which represents about 17% and 16% of TRM6 and TRM61’s total surface area, respectively. As shown in Fig. 2, TRM6 mainly interacts with TRM61 through four major sites. For site A, the C-terminal helix α11 of TRM61 inserts into a channel formed by the N-terminal loop and helix α1 of TRM6. Residues Val^374^ and Arg^369^ of TRM61 make hydrophobic interactions with residues Met^1^, Phe^9^, Ile^47^ and Tyr^49^ of TRM6 (Fig. 2A). In addition, the side-chain atom NH1 of Arg^369^ (TRM61) contributes two hydrogen bonds with the main-chain carbonyl of residues Asn^44^ and Ile^47^ (TRM6), respectively (Fig. 2A). For site B, the “n” shape loop following strands β13 of TRM6 makes close contact with the concave surface enclosed by strand β12 and α9 of TRM61. The interface involves a hydrophobic interaction, including residues Tyr^422^ (TRM6), Arg^426^ (TRM6), Leu^429^ (TRM6), Ile^233^ (TRM61), Val^236^ (TRM61), Leu^240^ (TRM61), Met^253^ (TRM61), and Tyr^357^ (TRM61) (Fig. 2B). Beyond that, the side-chain atom OE2 of Glu^255^ (TRM61) forms two hydrogen bonds with the side-chain atoms NH2 and NE of residue Arg^420^ (TRM6) (Fig. 2B). The side-chain atoms NE and NH2 of Arg^426^ (TRM6) contribute another two hydrogen bonds with the side-chain atoms OD1 and OD2 of Asp^237^ (TRM61), respectively (Fig. 2B).

For site C, the strand β12 of TRM6 and the strand β13 of TRM61 are antiparallel to each other, forming a β-sheet that involves eight hydrogen bonds (Fig. 2C). For site D, the N-terminal β-barrel domain and helix α1 of TRM61 form extensive hydrophobic interactions with the Rossmann fold domain of TRM6. Residues Tyr^9^, Trp^22^, Ile^28^, His^74^, Leu^76^, Leu^82^, and Leu^85^ of TRM61 form a hydrophobic core packing with residues Tyr^237^, Pro^385^, Leu^410^, Trp^444^, His^446^, and Val^448^ of TRM6 (Fig. 2D). Additionally, the side-chain atom NH2 of Arg^384^ (TRM6) forms hydrogen bond with the main-chain carbonyl of residue Lys^10^ of TRM61 (Fig. 2D). The side-chain atoms NH1 and NH2 of Arg^25^ (TRM61) make another two hydrogen bonds with the side-chain atom OG of Ser^413^ and main-chain carbonyl of Pro^412^ of TRM6, respectively (Fig. 2D).

### Two TRM6-TRM61 heterodimers assemble as a heterotetramer

**Figure 3:**
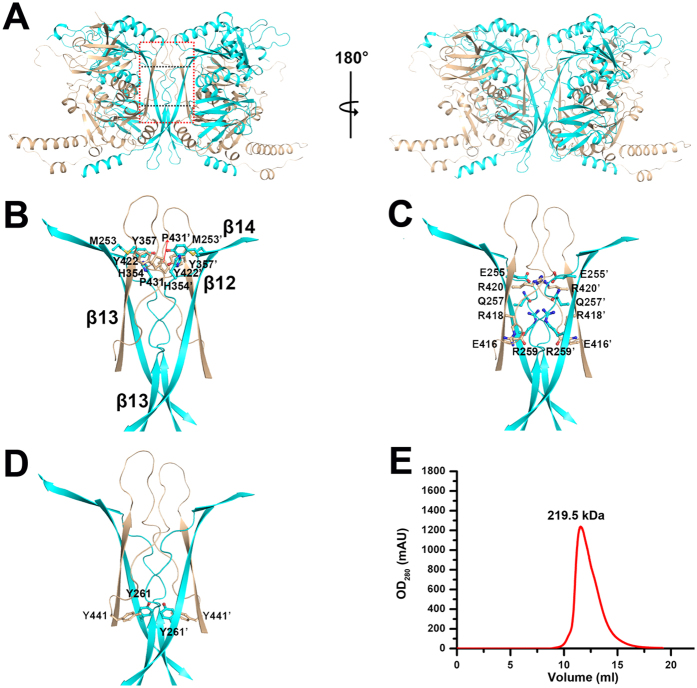
The heterotetramer interface of TRM6-TRM61. (**A**) Two views of the heterotetramer architecture of the TRM6 and TRM61 complex. The heterotetramer interface is mediated by a central β-barrel structure that consists of β13 (TRM6), loop β13/β14 (TRM6), β12 (TRM61) and loop β13/β14 (TRM61). The heterotetramer interface is marked with a red rectangle. Details show of the interface of the top (**B**), center (**C**) and bottom (**D**) of the β-barrel. The residues involved in the interaction are labeled and shown as sticks. (**E**) Gel-filtration analysis of the TRM6-TRM61 complex. Wild-type TRM6-TRM61 complex elutes with a molecular weight of approximately 219.5 kDa.

Previously, we have shown that a symmetric unit of the TRM6-TRM61 crystal contains one molecule of TRM6 and one molecule of TRM61, forming a 1:1 heterodimer. Further examination of symmetry- related molecules indicated that TRM6 and TRM61 form a 2:2 tetrameric heterocomplex, displaying a “Ω“ shape. As shown in Fig. 3A, two symmetry-related TRM6-TRM61 heterodimer come together to form a central β-barrel structure that consists of β13 (TRM6), loop β13/β14 (TRM6), β12 (TRM61) and loop β13/β14 (TRM61). The top of the barrel contains a hydrophobic core, formed by residues Tyr^422^ (TRM6), Pro^431^ (TRM6), Met^253^ (TRM61), His^354^ (TRM61), and Tyr^357^ (TRM61) (Fig. 3B). The center of the barrel is filled with numerous hydrophilic side-chains, including residues Glu^416^ (TRM6), Arg^418^ (TRM6), Arg^420^ (TRM6), Glu^255^ (TRM61), Gln^257^ (TRM61) and Arg^259^ (TRM61) (Fig. 3C). The bottom of the barrel consists of a cage of four tyrosine residues (Fig. 3D). To investigate the oligomer state of TRM6-TRM61 holoenzyme in solution, we performed the size-exclusion chromatography assay. As shown in Fig. 3E, the TRM6-TRM61 holoenzyme eluted with a molecular weight of approximately 219.5 kDa, which is very close to the theoretical value of 196.6 kDa for the TRM6-TRM61 heterotetramer. This result can confirm our structural observation and hints that *S. cerevisiae* TRM6-TRM61 may function as a heterotetramer.

### A TRM6-TRM61 heterotetramer constitutes two L-shaped tRNA binding regions

**Figure 4:**
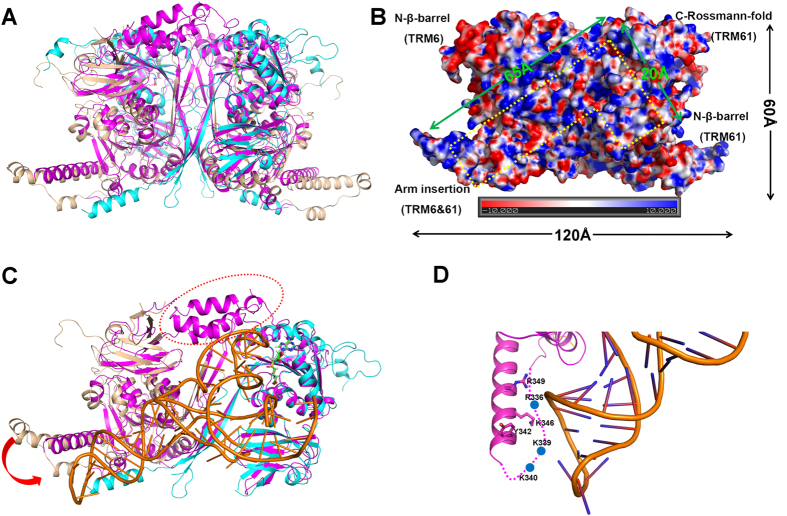
A possible L-shaped tRNA binding region of TRM6-TRM61. (**A**) Superposition of *S. cerevisiae* TRM6-TRM61 heterotetramer with the *H. sapiens* homologue. The *S. cerevisiae* TRM6 and TRM61 are colored the same as Fig. 1A. The *H. sapiens* TRM6-TRM61 complex is colored in magenta. (**B**) An electrostatic potential view of the TRM6-TRM61 complex. The electrostatic surface is calculated in *PyMOL* using APBS. A possible L-shaped tRNA binding region is shown as a dashed line. (**C**) Superposition of *S. cerevisiae* TRM6-TRM61 with the *H. sapiens* TRM6-TRM61-tRNA_3_^Lys^-SAM. The *S. cerevisiae* TRM6 and TRM61 are colored the same as Fig. 1A. The *H. sapiens* TRM6-TRM61 complex is colored in magenta, the tRNA_3_^Lys^ and SAM are shown as sticks and are colored in orange and green, respectively. Residues 88–155 of human TRM6 are marked by red dash line. (**D**) A close-up view of the region surrounding the anticodon arm of the tRNA_3_^Lys^ in *H. sapiens* TRM6.

In prokaryotes, four molecules of TrmI form a homotetramer, binding up to two molecules of target tRNA. Similar to the architecture of TrmI, two TRM6-TRM61 heterodimers assemble into a heterotetramer in *H. sapiens*, which constitute two identical substrate tRNA binding regions^24^. Each one is enriched with positively charged residues that come together to form an L-shape. Combining the structural superimposition and sequence alignment results, we found that the *S. cerevisiae* TRM6-TRM61 heterotetramer displays a similar fold as the *H. sapiens* homologue, and the residues involved in interaction with the substrate tRNA are highly conserved (Figs 4A and S1–2). As shown in Fig. 4B, the N-terminal β-barrel domain of TRM61, C-terminal Rossmann-fold domain of TRM61, N-terminal β-barrel domain of TRM6 and the arm insertions of TRM6-TRM61 enclose a cleft with 65 Å in length and 20 Å in width. The dimension of this cleft seems large enough to accommodate a double-helical RNA, and is a good candidate for accommodating the anticodon arm of target tRNA (Fig. 4B). In addition, considering the substrate A58 located at the TψC-loop is required to insert into the catalytic center composed by the C-terminal Rossmann-fold domain of TRM61. We speculate that the acceptor arm of the substrate tRNA mostly protrudes toward the N-terminal β-barrel domain of TRM61 (Fig. 4B).

The complex structure of *H. sapiens* TRM6-TRM61-tRNA_3_^Lys^-SAM resolved by Stroud and his colleague could assist us docking a model for further understanding of the substrate tRNA recognition mechanism of *S. cerevisiae* TRM6-TRM61. In light of the structure of *H. sapiens* TRM6-TRM61-tRNA_3_^Lys^-SAM, we could realize that the substrate tRNA is refolded upon binding to TRM6-TRM61. The D-loop that tightly associates with the TψC-loop is driven away from its buried position, and makes direct interactions with the N-terminal β-barrel domain of TRM61. Thus, the adenine at position 58 located in the TψC-loop could be accessible to the active site. Given the structural similarity between *S. cerevisiae* and *H. sapiens* TRM6-TRM61, we speculate that this large conformational changes may also associate with the interaction between *S. cerevisiae* TRM6-TRM61 and its target tRNA.

### The SAM binding site and a possible adenine-binding pocket

**Figure 5:**
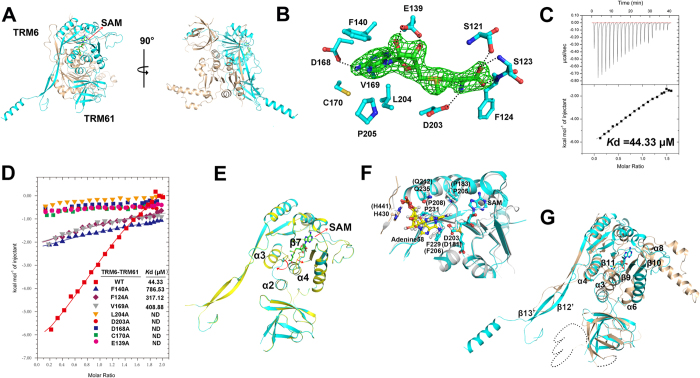
The SAM binding site and a possible adenine-binding pocket of TRM6-TRM61. (**A**) Two views of the *S. cerevisiae* TRM6-TRM61 holoenzyme in complex with SAM. (**B**) SAM binding model of *S. cerevisiae* TRM6-TRM61 holoenzyme. The Fo-Fc difference electron density map (contoured at 3.0σ) for SAM is shown as green. The residues involved in interacting with SAM of TRM6-TRM61 holoenzyme are labeled and colored cyan. The dashed lines represent hydrogen bonds. (**C**) ITC profile of SAM titrated against wild-type TRM6-TRM61. (**D**) ITC fitting curves of SAM to TRM6-TRM61 and its mutants. (**E**) The conformational change in the active site of TRM6-TRM61 holoenzyme upon SAM binding. The movements of the key structural elements are highlighted by arrows. The apo and SAM-bound structures of TRM61 are colored in yellow and cyan, respectively. (**F**) Structural superposition of the active site of *S. cerevisiae* TRM61 with its *H. sapiens* counterpart. The *S. cerevisiae* and *H. sapiens* TRM61 are colored the same to Fig. 4B. Adenine at position 58 is shown as stick and is colored in yellow. (**G**) Structural superposition of the *S. cerevisiae* TRM6 with TRM61. The TRM6 and TRM61 are colored the same to Fig. 1A. The segment that is invisible in the electron density map is shown as a dashed line.

To decipher the methyl donor recognition model of the two two-subunit tRNA m^1^A58 methyltransferase TRM6- TRM61, we determined the structure of *S. cerevisiae* TRM6-TRM61 in complex with SAM at 2.2 Å. The electron density map clearly shows the presence of a bound SAM molecule in a TRM6-TRM61 holoenzyme. As shown in Fig. 5A, SAM is situated in a cleft on the surface of the C-terminal Rossmann-fold domain of TRM61. The interaction between TRM61 and SAM can be divided into three parts in accordance to the moieties of SAM. For the adenine moiety, the side-chain atom OD1 of Asp^168^ makes a hydrogen bond with the nitrogen N6 of the adenine (Fig. 5B). In addition to this hydrogen bond, hydrophobic residues Phe^140^, Val^169^, Cys^170^, Leu^204^, and Pro^205^ also make extensive van der Waals interactions with the adenine ring (Fig. 5B). For the ribosyl moiety, the side chain of Glu^139^ forms two hydrogen bonds with the O2′ and O3′ hydroxyl groups (Fig. 5B). The interaction between TRM61 and the homocysteine moiety of SAM is dominated by four hydrogen bonds. The side-chain atom OD2 of Asp^203^ contributes to the first hydrogen bond with the amino group of the homocysteine (Fig. 5B). The carboxyl group of the homocysteine makes another three hydrogen bonds with the main-chain amide of Phe^124^, main-chain amide of Ser^123^, and side-chain atom OG of Ser^121^, respectively (Fig. 5B). To confirm our structural observation, we performed the ITC experiments to investigate whether these residues are vital for interacting with SAM. We constructed eight mutants TRM6-TRM61^F124A^, TRM6-TRM61^E139A^, TRM6-TRM61^F140A^, TRM6-TRM61^D168A^, TRM6-TRM61^V169A^, TRM6-TRM61^C170A^, TRM6- TRM61^D203A^ and TRM6-TRM61^L204A^. The ITC experiments show that the binding affinity of wild-type TRM6-TRM61 holoenzyme to SAM is 44.33 ± 4.55 μM, whereas these mutants completely or largely abolish the binding ability (Fig. 5C,D and Table 2). These results could well support our structural observation.

Superimposition of the structures of TRM61 in the presence and absence of SAM gives an r.m.s.d of 0.154 Å for 571 comparable Cα atoms. The overall structure of TRM61 in complex with SAM closely resembles that of the apo form (Fig. 5E), and the largest variation mainly occurs in the loop β7/α4 and loop α2/α3. In the structure of apo TRM61, loop β7/α4 and loop α2/α3 lock the binding pocket for the homocysteine moiety of SAM. Upon SAM binding, loop β7/α4 and loop α2/α3 move away from the SAM binding pocket, which generates sufficient space to suit its entrance (Fig. 5E). These two structures provide a structural basis for understanding the conversion of the SAM binding pocket from a closed to an open state, and indicate that the binding of SAM to TRM6-TRM61 holoenzyme occurs through an induced-fit mechanism.

Up until now, numerous structures of tRNA methyltransferases in complex with substrate tRNAs have been reported, including the *Escherichia coli* m^5^U54 tRNA methyltransferase TrmA and the *archaeal* m^1^G37 tRNA methyltransferases Trm5^25^,^26^. Though they share a low sequence homology and distinct structural features, these enzymes utilize a similar strategy to accommodate the target base. As shown in TrmA, the U54 base inserts into a hydrophobic pocket formed by residues Phe^188^, Pro^191^, Pro^301^, and Phe^351^, which is adjacent to the methyl donor SAM^25^. Additional, residues Gln^190^, Asp^299^, and Gln^358^ make five hydrogen bonds to further stabilize the U54 base^25^. For Trm5, residues Val^140^, Arg^145^, Tyr^177^, Asn^265^, Leu^266^, Pro^267^, and Try^320^ constitute the G37 base binding center, situating it close to the methyl group of the SAM to facilitate the reaction^26^. As expected, adjacent to the methyl donor binding site of TRM6-TRM61 holoenzyme in *S. cerevisiae*, residues Gln^92^ (TRM61), Asp^203^ (TRM61), Pro^205^ (TRM61), Phe^229^ (TRM61), Pro^231^ (TRM61), Gln^235^ (TRM61), His^430^ (TRM6), and Met^433^ (TRM6) form a pocket, which is optimal to accommodate the A58 of a target tRNA (Fig. 5F). In the structure of *H. sapiens* TRM6-TRM61-tRNA_3_^Lys^-SAM, the corresponding position is just in charge of accommodating the base A58 of tRNA_3_^Lys^ (Fig. 5F). This feature proposes the evolutionary conservation of tRNA m^1^A58 methyltransferase between *S. cerevisiae* and *H. sapiens*.

### Structural insights into the evolutionary relationship between TRM6 and TRM61

Studies of the mutants involved in *GCN4* translational control in *S. cerevisiae* allowed to identify a newly characterized two-subunit tRNA m^1^A58 methyltransferase TRM6-TRM61, which was encode by two non-identical genes *TRM6* and *TRM61*, respectively^21^,^22^. The interest in studying the TRM6-TRM61 holoenzyme in eukaryotes is due to the fact that none of the prokaryotic genomes sequenced so far contains an ortholog of gene *TRM6*, which has been proved to be essential for the tRNA m^1^A58 methyltransferase activity both *in vitro and in vivo*. In this study, we solved the structure of TRM6-TRM61 and TRM6-TRM61 in complex with methyl donor SAM from *S. cerevisiae*. TRM6 and TRM61 have a similar core structure, both adopting a TrmI-like fold. The r.m.s.d between TRM6 and TRM61 is 8.637 Å for 56 comparable Cα atoms. Structural comparison between TRM6 and TRM61 shows numerous marked variations. The first significant difference is the distinct composition of the N-terminal β-barrel domain. As shown in Fig. 5G, the β-barrel domain of TRM61 is composed of a short helix and three hairpin β-motifs. In TRM6, it consists of a short helix, seven antiparallel β strands and a highly flexible region enriched in positively charged residues. The second significant difference is found in the methyl donor binding region. Compared with TRM61, segments β9/α6 and β10/α8 of TRM6 move away from the SAM binding site (Fig. 5G). Instead, Segment α3/α4 moves toward the active center and makes clash with the homocysteine moiety of SAM (Fig. 5G). These conformation changes finally disrupt the SAM binding pocket and cause TRM6 lost the SAM binding ability. The third significant difference is that TRM61 has a sequence insertion between β12 and β13, which is enriched in positively charged residues and may be involved in the substrate tRNA recognition (Fig. 5G). In addition, there is an arm insertion between β10 and β11 in TRM6, which protrudes from the core (Fig. 5G). Taken together, our results support the note that eukaryotic *TRM6* and *TRM61* may evolve by gene duplication from prokaryotic *TrmI*. During the divergent evolution, TRM6 lost the catalytic residues and methyl donor binding ability. However, TRM6 and TRM61 added more structural elements to better fulfill the tRNA binding role in eukaryotes.

### The difference between *S. cerevisiae* TRM6-TRM61 and its prokaryotic counterpart TrmI

The mainly difference between *S. cerevisiae* TRM6-TRM61 and its prokaryotic counterpart TrmI is the distinct strategy for recognizing the target tRNA. In prokaryotes, TrmI functions as a homotetramer, with two tRNA binding grooves and four identical catalytic centers. Distinctly different from the prokaryotic TrmI, the eukaryotic m^1^A58 tRNA methyl- transferases consists of two subunits, TRM6 and TRM61. Though TRM6 and TRM61 adopt a similar TrmI-like fold, they evolve more structural elements for recognizing the target tRNA. Moreover, TRM6 loses the methyl donor binding ability, thus one TRM6-TRM61 heterotetramer only possesses two catalytic centers. These structural differences between TRM6-TRM61 and TrmI hint that the substrate tRNA recognition mechanism of eukaryotic tRNA (m^1^A58) methyltransferase may be more complicated and the catalytic reaction may be more accurate than that of its prokaryotic counterpart.

### Structural comparison with *H. sapiens* TRM6-TRM61 indicates a hand open-close substrate tRNA recognition strategy

The human homolog of the yeast tRNA m^1^A58 methyltransferase was identified through amino acid sequence identity and complementation of the yeast temperature-sensitive *TRM6* and *TRM61* mutant phenotypes^27^. When co- expressed in yeast, the *H. sapiens* TRM6-TRM61 could catalyze the *in vitro* methyl transfer reaction for both the yeast initiator tRNA_i_^Met^ and human tRNA_3_^Lys ^27. Recently, the structure of *H. sapiens* TRM6-TRM61-tRNA_3_^Lys^-SAM was report by Stroud’s group^24^. This structure firstly discloses the substrate tRNA recognition model of tRNA m^1^A58 methyltransferase in eukaryotes. According to this complex structure, we could find that the substrate tRNA undergoes a large conformation change upon binding to TRM6-TRM61 holoenzyme. However, due to a lack of the structure of apo TRM6-TRM61 holoenzyme, what kind of conformation change would happen in TRM6-TRM61 holoenzyme during the tRNA recognition process is still elusive. In the structure of apo *S. cerevisiae* TRM6-TRM61, the residues 88–188 located at the N-terminal β-barrel domain of TRM6 are not visible due to the flexibility. Interestingly, this region covers at the tRNA binding pocket, and plays a vital role for interacting with the TψC-loop of substrate tRNA in the structure of *H. sapiens* TRM6-TRM61-tRNA_3_^Lys^ (Fig. 4C). In addition, the arm insertion between β10 and β11 of TRM6 in the apo *S. cerevisiae* TRM6-TRM61 protrudes away from the position that is suitable for recognizing the anticodon arm of the substrate tRNA. In the structure of *H. sapiens* TRM6-TRM61-tRNA_3_^Lys^ complex, the corresponding region is enriched with positive charged residues, and these residues are also conserved between the *S. cerevisiae* and *H. sapiens* (Fig. 4D).Taken together, we propose a hand open-close mechanism for eukaryotic tRNA m^1^A58 methyltransferase to recognize the target tRNA. In the tRNA-unbound state, the TRM6-TRM61 holoenzyme likes an open hand, which creates enough space for the entrance of target tRNA. Then, the open hand converts to a closed form to lock the substrate tRNA in a position suitable for catalysis.

## Materials and Methods

### Gene cloning, protein expression and purification. Gene cloning, protein expression and purification

Full-length TRM6 and TRM61 from *S. cerevisiae* was cloned as described previously^28^. We optimized the condition for expression and purification. Briefly, the bacteria expressing the recombinant proteins was cultured in LB medium at 37 °C to OD600 = 0.8. Then, the bacteria was cooled to 16 °C, and induced with 0.5 mM IPTG. After growing for approximately 16 h at 16 °C, the cells were harvested and disrupted by sonication in buffer A (50 mM Tris-HCl PH 7.5, 300 mM NaCl and 5% glycerol). The supernatant lysate was loaded onto a Ni^2+^-NTA column (GE Healthcare, USA) pre-equilibrated with buffer A supplemented with 10 mM imidazole. The recombinant TRM6-TRM61 complex was eluted with 20 ml buffer A supplemented with 200 mM imidazole. Then, the protein was further purified using Superdex 200 (GE Healthcare, USA) gel-filtration chromatography equilibrated with buffer B (20 mM Tris-HCl PH 8.0, 300 mM NaCl) supplemented with 5 mM dithiothreitol. The fractions corresponding to the peak were pooled and concentrated, and stored at −80 °C for further steps. TRM6-TRM61 mutants were generated by PCR with the MutanBEST Kit (TaKaRa) using the parent expression plasmid pETDuet-TRM6-TRM61 as template. The mutant plasmids were then confirmed by DNA sequencing (Invitrogen). The TRM6-TRM61 mutant proteins were overexpressed and purified as described for the wild-type TRM6- TRM61. A selenomethionine derivative of TRM6-TRM61 was overexpressed in the same competent cells as native TRM6-TRM61 but using M9 medium based on a methionine- biosynthesis inhibition method. The purification of Se-TRM6-TRM61 followed the same protocol as used for native TRM6-TRM61.

### Crystallization, Data Collection, and Structure Determination

The crystallization of apo TRM6-TRM61 has been described previously^28^. Native and SeMet-derivative TRM6-TRM61 were concentrated to ~15 mg/ml in buffer B supplemented with 5 mM dithiothreitol before crystallization. The TRM6-TRM61-SAM complex was prepared by mixing the protein with a three-fold molecular excess of S-adenosyl-L-methionine. Crystals suitable for X-ray diffraction were all grown at 293K via the sitting-drop vapour-diffusion method with the mother liquor containing 0.1 M HEPES, PH 7.5, 2% v/v (+/−)-2-Methyl-2, 4-pentanediol, 10% w/v Polyethylene glycol 6000 for 3 days. Before data collection, the crystals were quick- soaked in a cryoprotectant solution consisting of respective reservoir solution supplemented with 30% (v/v) glycerol and then flash-cooled in a nitrogen stream at 100 K. X-ray diffraction datas were collected on beamline 17U1 of Shanghai Synchrotron Radiation Facility (SSRF). The data were processed and scaled with HKL-2000^29^ and programs from the CCP4 package^30^. The structure of TRM6-TRM61 was determined through the single- wavelength anomalous dispersion (SAD) phasing technique with the selenium anomalous signal using the *Autosol* program implemented in *PHENIX*^31^. The initial model was built automatically using the program *Autobuild* in *PHENIX*^31^. Using the TRM6-TRM61 structure as the search model, the structure of TRM6-TRM6–SAM was determined through the molecular replacement method using the program *MOLREP*^32^ implemented in *CCP4i*^30^. All of the initial models were refined using the maximum likelihood method implemented in *REFMAC5*^33^ as part of the *CCP4i*^30^ program suite and rebuilt interactively using the program *COOT*^34^. The final models were evaluated with the programs *MolProbity*^35^ and *PROCHECK*^36^. The crystallographic parameters are listed in Table 1. All of the structures in the figures were prepared with *PyMOL* (DeLano Scientific).

### Size-exclusion chromatography assay

A Superdex 200 column (10/300 GL; GE Healthcare) was used to estimate the apparent molecular mass of the TRM6-TRM61 holoenzyme from *S. cerevisiae*. Briefly, the samples of target protein or molecular-mass standards were loaded onto the column at a flow rate of 0.5 ml min^−1^ and eluted with 20 mM Tris-HCl pH 8.0, 300 mM NaCl, 5 mM DTT. The standard proteins (GE Healthcare) used in this study were β-amylase (200.0 kDa), alcohol dehydrogenase (150.0 kDa), albumin (66.0 kDa), carbonic anhydrase (29.0 kDa) and cytochrome c (12.4 kDa). The blue dextran (GE Healthcare) was used for void volume determination.

### Isothermal Titration Calorimetry

ITC assays were carried out using a MicroCal iTC200 calorimeter (GE Healthcare) at room temperature with 40 ul of 1 mM SAM in the injector cell and 260 ul of 0.1 mM wild-type TRM6-TRM61 and its mutants in the sample cell, respectively. The buffer for proteins and SAM was 20 mM Tris-HCl pH 8.0, 300 mM NaCl, 1 mM TCEP (Tris-(2-carboxyethyl) -phosphine hydrochloride). Twenty microliters injection volumes were used for all experiments. Two consecutive injections were separated by 2 min to reset the baseline. A reference measurement (SAM injected into the buffer) was carried out to compensate for the heat of dilution of SAM. ITC data was analyzed with a single-site fitting model using Origin 7.5 (OriginLab) provided by the manufacturer.

## Additional Information

**Accession codes:** The atomic coordinates and structure factors have been deposited in the Protein Data Bank with the accession codes: 5EQJ and 5ERG.

**How to cite this article**: Wang, M. *et al*. Crystal structure of the two-subunit tRNA m^1^A58 methyltransferase TRM6-TRM61 from *Saccharomyces cerevisiae*. *Sci. Rep.* **6**, 32562; doi: 10.1038/srep32562 (2016).

## Supplementary Material

Supplementary Information

## Figures and Tables

**Table 1:** Data collection and Refinement Statistics.

	TRM6-TRM61-SeMet	TRM6-TRM61	TRM6-TRM61-SAM
Data collection statistics			
Space Group	*P*3_2_21	*P*3_2_21	*P*3_2_21
Unit Cell Parameters			
*a*, *b*, *c* (Å)	140.5, 140.5, 102.9	138.7, 138.7, 102.7	139.9, 139.9, 103.5
*α*, *β*, *γ* (˚)	90.0, 90.0, 120.0	90.0, 90.0, 120.0	90.0, 90.0, 120.0
Wavelength (Å)	0.9785	0.9792	0.9785
Resolution limits (Å)^a^	50.00-2.70 (2.80-2.70)	50.00-2.20 (2.28-2.20)	50.00-2.20 (2.28-2.20)
Completeness (%)	100.0 (100.0)	99.8 (99.8)	99.9 (99.8)
Redundancy	11.1 (10.7)	8.3 (7.9)	7.7 (7.9)
*R*_merge_ (%)^b^	12.8 (42.9)	8.6 (70.6)	8.6 (77.0)
*R*_p.i.m_ (%)	4.0 (13.7)	3.2 (26.1)	3.3 (29.0)
Mean I/σ (I)	27.8 (5.1)	23.5 (3.4)	21.6 (2.4)
Refinement Statistics			
Resolution limits (Å )		50.00-2.20	50.00-2.20
No. of reflections		57895	59326
*R*_work_ (%)^c^/*R*_free_ (%)^d^		18.32/21.17	18.42/21.36
R.m.s.d for bonds (Å)		0.008	0.009
R.m.s.d for angles (˚)		1.129	1.120
B factor (Å^2^)			
Protein		50.45	49.75
Water		49.99	48.95
SAM			58.20
No. of non-hydrogen protein atoms		5297	5355
No. of water oxygen atoms		199	199
Ramachandran plot (%)			
most favored regions		92.3	91.9
additional allowed regions		7.7	8.1
PDB code		5EQJ	5ERG

^a^Values in parentheses are for the highest-resolution shell.

^b^*R*_*merge*_ = ∑|I_i_- <I> |/∑|I|, where I_i_ is the intensity of an individual reflection and <I> is the average intensity of that reflection.

^c^*R*_*work*_ = ∑||F_o_|-|F_c_||/∑|F_o_|, where F_o_ and F_c_ are the observed and calculated structure factors for reflections, respectively.

^d^*R*_*free*_ was calculated as *R*_*work*_ using the 5% of reflections that were selected randomly and omitted from refinement.

**Table 2:** The thermodynamic parameters of the ITC experiments.

Proteins	∆H kcal/mol	∆S cal/mol/deg	*K*_D_ μM	N
WT	−7.83	−6.31	44.33 ± 4.55	1.03
TRM61-TRM61^F124A^	−8.47	−12.4	317.12 ± 34.71	1*
TRM61-TRM61^E139A^			ND	
TRM61-TRM61^F140A^	−19.30	−50.5	786.53 ± 64.51	1*
TRM61-TRM61^D168A^			ND	
TRM61-TRM61^V169A^	−10.01	−18.0	408.88 ± 74.66	1*
TRM61-TRM61^C170A^			ND	
TRM61-TRM61^D203A^			ND	
TRM61-TRM61^L204A^			ND	

^*^Due to the low binding affinities of these interactions (C value < 1), we fitted the titration curves with “N” value fixed to 1, which could give more reasonable *K*_*D*_ values. Note that, in these fittings, △H might not be well determined^37^.
