# Supplementary material for: Crystal structure of the two-subunit tRNA m1A58 methyltransferase TRM6-TRM61 from Saccharomyces cerevisiae
Source: Sci Rep. 2016 Sep 1;6:32562. doi: 10.1038/srep32562 (PMC5007650; doi:10.1038/srep32562)
Supplement: Supplementary Information [file srep32562-s1.doc]

Crystal structure of the two-subunit tRNA m^1^A58 methyltransferase TRM6-TRM61 from *Saccharomyces cerevisiae*

Mingxing Wang^1,2,+^, Yuwei Zhu^1,2,+^, Chongyuan Wang^1,2^, Xiaojiao Fan^1,2^, Xuguang Jiang^1,2^, Mohammad Ebrahimi^1,2^, Zhi Qiao^1,2^, Liwen Niu^1,2^, Maikun Teng^1,2,*^ and Xu Li^1,2,*^

^1^ Hefei National Laboratory for Physical Sciences at Microscale, Innovation Center for Cell Signalling Network, School of Life Science, University of Science and Technology of China, Hefei, Anhui, 230026, People's Republic of China

^2^ Key Laboratory of Structural Biology, Hefei Science Center of CAS, Chinese Academy of Science, Hefei, Anhui, 230026, People's Republic of China.

^*^Correspondence and requests for materials should be addressed to Xu Li (email: sachem@ustc.edu.cn) or Maikun Teng (email: mkteng@ustc.edu.cn)

^+^These authors contributed equally to this work.

**Supplementary Figures**


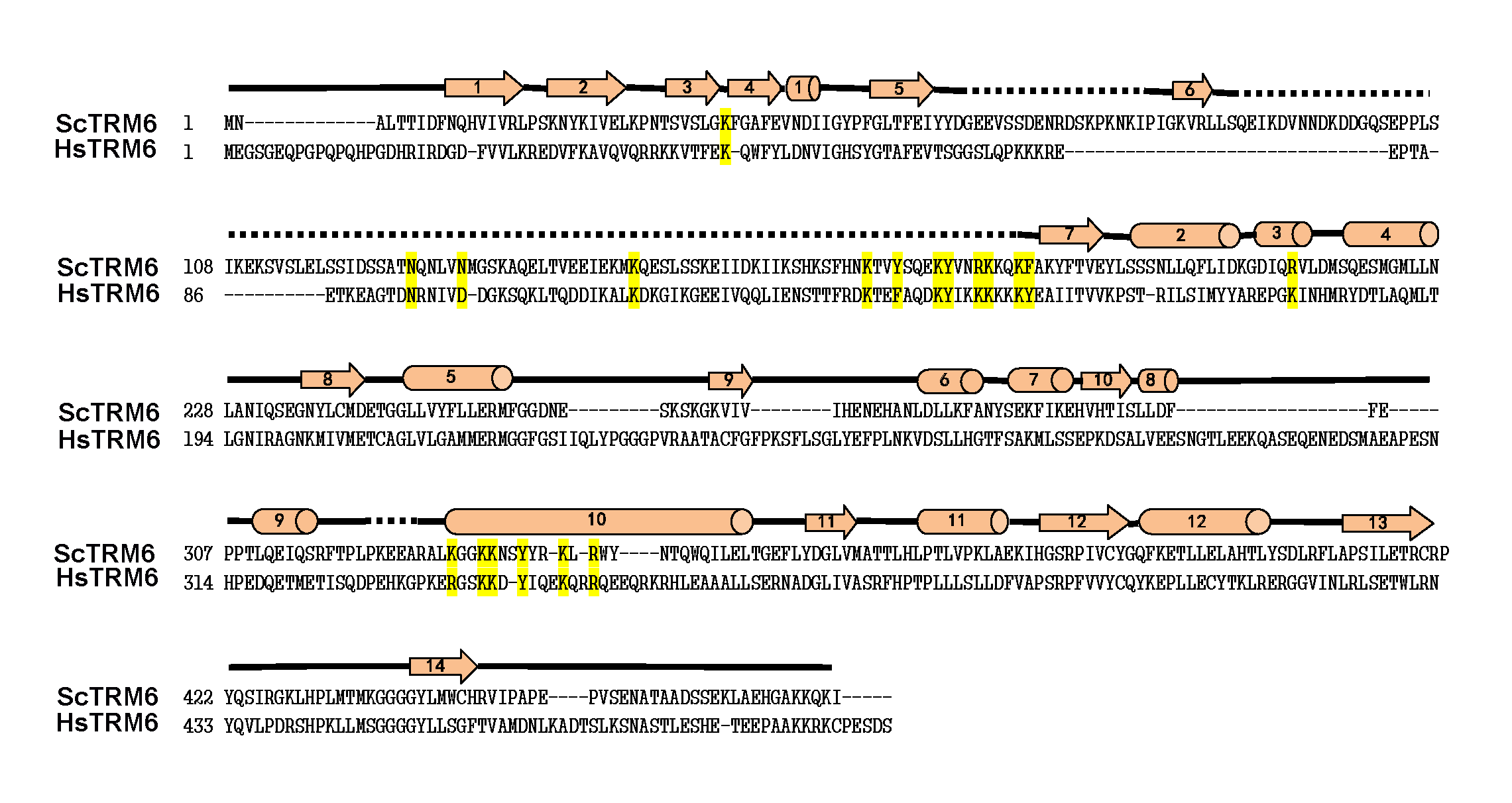
**Figure S1**

**Figure S1** Sequence alignment of *S. cerevisiae* TRM6 with the *H. sapiens* homologue. Secondary structural elements of *S. cerevisiae* TRM6 are shown above the sequences. The conserved residues involved in interaction with tRNA_3_^Lys^ are colored in yellow.


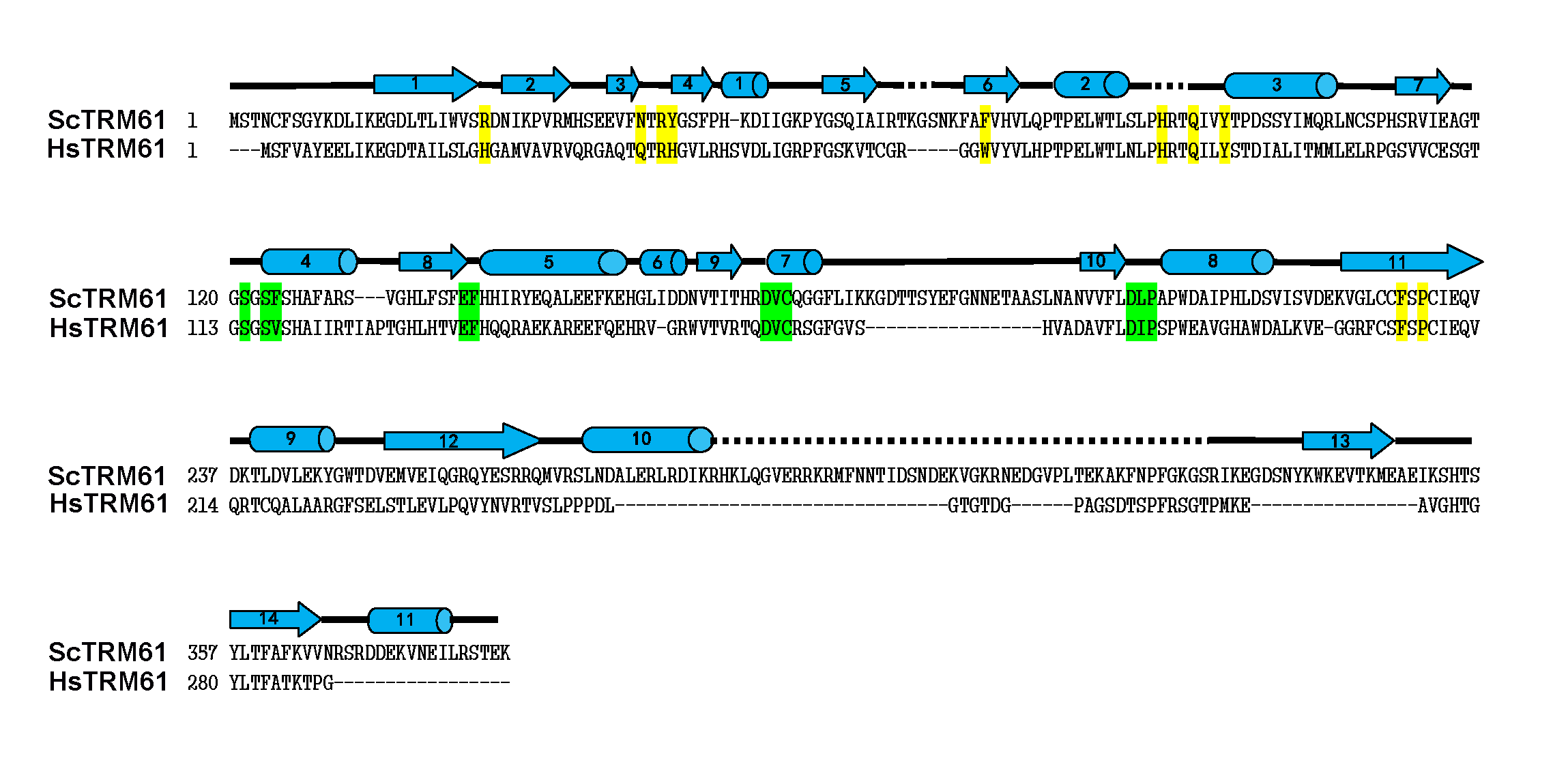
**Figure S2**

**Figure S2** Sequence alignment of *S. cerevisiae* TRM61 with the *H. sapiens* homologue. Secondary structural elements of *S. cerevisiae* TRM61 are shown above the sequences. The conserved residues involved in interaction with tRNA_3_^Lys^ are colored in yellow, and the residues involved in interaction with SAM are colored in green.
